# A Pesky Spleen: A Painful Presentation of Extrapulmonary Sarcoidosis

**DOI:** 10.7759/cureus.43077

**Published:** 2023-08-07

**Authors:** Hania M Woomer, Teseir N Hamdani, Vincent Graffeo, Bisher Mustafa

**Affiliations:** 1 Internal Medicine, Marshall University Joan C. Edwards School of Medicine, Huntington, USA; 2 Pathology, Marshall University Joan C. Edwards School of Medicine, Huntington, USA

**Keywords:** noncaseating granulomas, sarcoidosis, splenectomy, clinical manifestations, splenic sarcoidosis

## Abstract

Sarcoidosis is an inflammatory and granulomatous disease of uncertain etiology that can impact various organ systems and exhibits diverse clinical presentations, which adds to the complexity of disease diagnosis and management. Pathologically, it is distinguished by the presence of noncaseating granulomas within the affected organ system. In this case report, we describe a 34-year-old Caucasian female patient with isolated splenic and possible hepatic involvement of sarcoidosis, presenting with severe abdominal pain. The absence of the typical pulmonary, cutaneous, or joint involvement posed challenges in achieving a definitive diagnosis and determining the appropriate management. Imaging studies revealed hepatic and splenic hypodensities, necessitating consideration of various differential diagnoses, including lymphoproliferative disorders, immunological disorders, environmental particle exposure, infectious causes, neoplasms, and drug reactions. The severity of symptoms in this case required hospital admission for pain and nausea control, biopsy, and eventual splenectomy with pathology that confirmed the diagnosis of splenic sarcoidosis.

## Introduction

Sarcoidosis, a multisystem inflammatory disease, was initially identified and named over a century ago. In 1878, Sir Jonathan Hutchinson first described the condition of sarcoidosis [1]. The hallmark of sarcoidosis is the infiltration of affected organs by noncaseating granulomas. It is thought to be an aberrant immune response to an environmental antigen that results in this observed granulomatous formation [2]. While it can occur in individuals of any ethnicity and age, the highest incidence is observed among African Americans and Scandinavians, with most cases presenting between the ages of 25 and 40. A secondary peak in incidence is noted in women over the age of 50. The estimated annual incidence of sarcoidosis ranges between two and 11 cases per 100,000 people [3]. Notably, racial disparities exist in the incidence of sarcoidosis. A study conducted by a US Health Maintenance Organization, after adjusting for age, sex, area of residence, and study year, demonstrated an almost four-fold higher risk among African Americans as compared to Caucasians [4]. Sarcoidosis can follow a self-limiting course or progress to a chronic disease state. Common presenting symptoms include non-specific manifestations, with fatigue observed in over 70% of cases [5]. Lymphadenopathy has been reported in up to 20% of patients, affecting cervical, axillary, inguinal, and epitrochlear nodes. Additionally, general symptoms of sarcoidosis may encompass fever, night sweats, and weight loss, particularly in cases involving hepatic manifestations [6]. Although sarcoidosis can affect any organ, over 90% of patients with clinically significant sarcoidosis exhibit intrathoracic lymph node involvement, pulmonary system abnormalities, skin manifestations, and/or ocular complications. Lofgren syndrome is a clinically unique phenotype of sarcoidosis with overlapping features of some of the most common symptoms. It typically presents acutely with erythema nodosum, bilateral hilar lymphadenopathy, fever, and migratory polyarthritis, without granulomatous skin involvement [7]. Symptomatic splenic involvement is a rare presentation, being reported in only about 6% of cases within six months from the initial diagnosis [8]. Hypersplenism can lead to pain, anemia, leukopenia, and thrombocytopenia [9]. While no definitive diagnostic test exists for sarcoidosis, its diagnosis relies on the presence of clinical and radiographic manifestations consistent with the disease, the exclusion of alternative conditions, and histopathologic identification of noncaseating granulomas [10]. Clinically, sarcoidosis is primarily diagnosed through a process of exclusion due to its intricate, multiorgan nature and the absence of pathognomonic features [1]. Regular follow-up is recommended for individuals diagnosed with sarcoidosis to monitor systemic manifestations. In this case, we present an instance of suspected splenic and hepatic sarcoidosis based on imaging with two inconclusive biopsies. The condition was later confirmed through post-surgical pathology after the patient underwent a splenectomy. Prior to the splenectomy, the diagnosis remained unclear despite an extensive work-up and further evaluation at a tertiary care center. Although antibiotics were tried based on infectious disease recommendations, no definitive diagnosis had been made at that time. The patient endured persistent symptoms of abdominal pain for months, significantly impairing her quality of life. Despite receiving advice to return to the tertiary care center for further evaluation, the patient elected for a splenectomy due to the severity of her pain. The case was thoroughly discussed, and the multidisciplinary team reached an agreement that a splenectomy was acceptable as the next course of action based on this patient and her specific clinical situation.

This article was previously presented as an abstract at the 2023 WV Society of Hospital Medicine Abstract Competition in May 2023.

## Case presentation

**Figure 1:**
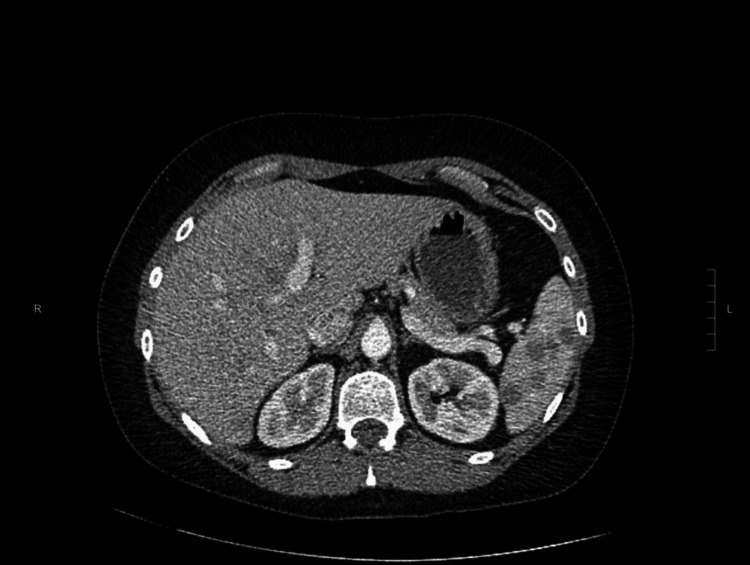
Computed tomography of the abdomen and pelvis with IV contrast showing low-attenuation lesions in the liver and spleen

**Figure 2:**
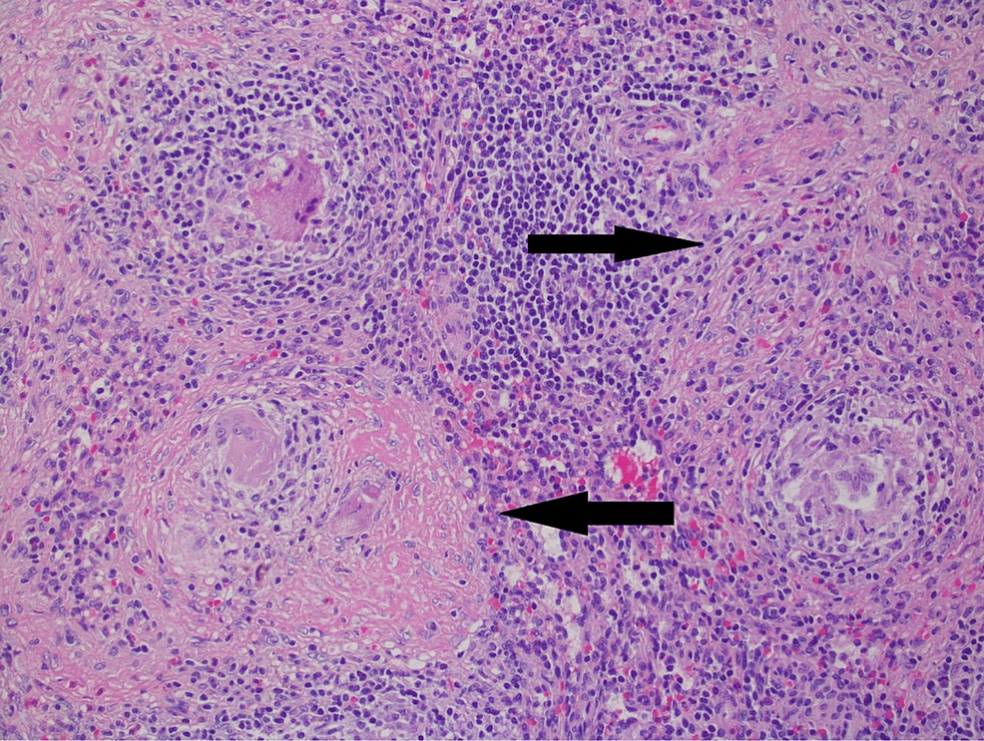
Post-splenectomy pathology with non-necrotizing granuloma with multinucleated cells and fibrosis The arrows point to granulomas composed of epithelioid histiocytes. Hematoxylin and eosin, original magnification 400X.

A 34-year-old Caucasian female with a medical history of anxiety, depression, insomnia, and migraines presented with acute left upper quadrant abdominal pain that abruptly awakened her from sleep, accompanied by symptoms of nausea and vomiting. She denied cough, shortness of breath, chest pain, changes in visual acuity, rash, alterations in bowel or bladder function, unintentional weight loss, or recent illness. Her current medication regimen consisted of drospirenone/ethinyl estradiol oral contraceptive, clonazepam, melatonin, and zaleplon. Regarding her social history, she reported tobacco use and employment at a local pet store. Upon presentation, she was hemodynamically and vitally stable. The physical examination revealed severe tenderness upon palpation of the left upper quadrant while initial laboratory investigations, encompassing electrolytes, calcium level, liver function studies, and urine analysis were within normal reference ranges. The complete blood count demonstrated no abnormalities, except for a slightly elevated eosinophil count of 6.2% (reference range 0-5%). Contrast-enhanced abdominal and pelvic computed tomography (CT) displayed multiple low-attenuation lesions in the liver and spleen (Figure 1). A liver biopsy, guided by CT, was performed due to concerns regarding infectious or malignant etiologies, and the biopsy was inconclusive. Subsequently, universal polymerase chain reaction (PCR) analysis was conducted on the biopsy, indicating the presence of nonspecific bacterial DNA and providing indeterminate results. The patient underwent an extensive infectious workup, including tests for *Cytomegalovirus*, Human immunodeficiency virus, Epstein-Barr virus, hepatitis panel, acid-fast bacilli, *Bartonella*, *Chlamydia trachomatis*, *Chlamydia psittaci*, *Treponema pallidum*, enteric bacteria profile, Lyme disease, *Coxiella*, *Cryptococcus*, *Histoplasma capsulatum*, *Blastomyces dermatitidis*, ova and parasites, and *Toxoplasma gondii*. Additionally, an autoimmune workup was conducted, involving testing for systemic lupus erythematosus, mixed connective tissue diseases, Sjogren's syndrome, scleroderma, polymyositis, alpha-1 antitrypsin, and ceruloplasmin. The comprehensive workup yielded unremarkable results, except for an elevated C-reactive protein level of 4.2 mg/dL (reference range <0.9 mg/dL). Additional imaging studies, including CT of the chest and CT of the head, did not reveal any significant findings. The patient received symptomatic management and was referred to a tertiary care facility for further workup, which was also unrevealing. Despite extensive workup and symptomatic management, she continued to have persistent abdominal pain. She was then admitted to the hospital, where a repeat liver biopsy was performed via endoscopic ultrasound, this was inconclusive with some rare noncaseating granulomas. Universal PCR analysis was repeated, and the results were negative for fungal DNA or mycobacterium DNA. Sarcoidosis was suspected, prompting initial treatment with a prednisone taper. However, the patient did not tolerate the steroids, due to significant anxiety, agitation, and other adverse side effects. Although antibiotics were tried based on infectious disease recommendations, no definitive diagnosis had been made at that time, and this failed to provide relief for the patient. Consequently, the patient opted for splenectomy, and the subsequent pathology report confirmed the suspected diagnosis of sarcoidosis (Figure 2). Microbiological stains and cultures were negative. The patient remains asymptomatic.

## Discussion

Due to its intricate characteristics, the potential for multiorgan involvement, and the absence of pathognomonic features, sarcoidosis is primarily considered a diagnosis of exclusion. The diverse range of clinical presentations associated with sarcoidosis can present challenges in both diagnosing and managing the disease. While sarcoidosis was suspected in this case, the involvement of splenic and hepatic lesions with an inconclusive biopsy necessitated a comprehensive differential diagnosis, encompassing lymphoproliferative disorders, immunological disorders, environmental particles exposure, infectious causes, neoplasms, and drug reactions [3]. Extrapulmonary manifestations are observed in approximately 30% of sarcoidosis cases, with splenic and hepatic involvement occurring in only a small fraction, accounting for 6% and 11.5% of cases, respectively [8]. Instances of isolated splenic sarcoidosis without specific clinical or radiologic indications of pulmonary involvement are rare, with only a limited number of reported cases documented [10-14]. The management of sarcoidosis is challenging due to the variable clinical presentation, the decision on when and how to treat a patient is based on the risk of organ failure and quality of life. Glucocorticoids are the primary treatment for sarcoidosis, unfortunately, they are associated with dose-dependent side effects. Second-line treatment includes methotrexate, azathioprine, leflunomide, and mycophenolate [15]. In this specific case, the patient did not tolerate a trial of steroids and her symptoms of abdominal pain continued to affect her quality of life. Despite receiving advice to return to the tertiary care center for further evaluation and alternate treatment options, the patient refused to return to a tertiary care center and opted for a splenectomy due to the severity of her pain. After undergoing splenectomy, her pain resolved completely and the postoperative pathology report confirmed the suspected diagnosis of sarcoidosis.

## Conclusions

Isolated splenic sarcoidosis represents a rare manifestation of sarcoidosis, which poses challenges in terms of diagnosis due to the absence of a definitive diagnostic test. Instead, the diagnosis relies on the presence of clinical and radiographic manifestations consistent with sarcoidosis, exclusion of other diseases, and histopathologic identification of noncaseating granulomas. The treatment of sarcoidosis often involves the administration of steroids; however, in this specific instance, the patient exhibited poor tolerance, thereby requiring alternative therapeutic approaches. Improving care for patients with sarcoidosis often requires multidisciplinary teams working together to understand the unique patient presentation and affected organ system, to maximize the diagnosis and treatment for the safety and quality of life of the patient.
